# Immune Dysfunction Prior to *Staphylococcus aureus* Bacteremia Is a Determinant of Long-Term Mortality

**DOI:** 10.1371/journal.pone.0088197

**Published:** 2014-02-05

**Authors:** Jared A. Greenberg, Michael Z. David, Jesse B. Hall, John P. Kress

**Affiliations:** 1 Section of Pulmonary and Critical Care Medicine, Department of Medicine, University of Chicago, Chicago, Illinois, United States of America; 2 Section of Infectious Disease and Global Health, Department of Medicine, University of Chicago, Chicago, Illinois, United States of America; University of Cincinnati, United States of America

## Abstract

**Purpose:**

The clinical implications for patients who survive serious infections are not well understood. It has been hypothesized that the excess mortality for survivors of sepsis observed in epidemiological studies is due to increased vulnerability to subsequent infections. We undertook this study to identify characteristics of patients who are at high risk for death after surviving a common type of blood-stream infection.

**Materials and Methods:**

At a single academic medical center, 237 patients with *Staphylococcus aureus* bacteremia admitted during a three-year period were retrospectively identified. The primary outcomes were 30-day and 31 to 90-day mortality after the first positive blood culture. The primary predictor variable of interest was clinical immune dysfunction prior to bacteremia.

**Results:**

The 30-day mortality was not significantly different for patients with and without prior immune dysfunction. However, during days 31 to 90, 11 patients (20%) with prior immune dysfunction compared to 10 patients (8.6%) without prior immune dysfunction died (OR 2.59, 95% CI 1.03–6.53, p = 0.04). In a Cox-proportional hazard model controlling for age, there was a significant association between prior immune dysfunction and greater 31 to 90 day mortality (HR 2.44, 95% CI 1.01–5.90, p = 0.05) and a non-significant trend towards occurrence of subsequent infections and greater 31 to 90 day mortality (HR 2.12, 95% CI 0.89–5.07, p = 0.09).

**Conclusions:**

Patients with prior immune dysfunction are at high risk for death 31 to 90 days, but not <30 days, after *S. aureus* bacteremia. Further investigation is needed to determine if this finding is due to poor prognosis of chronic disease or increased vulnerability to subsequent infections.

## Introduction

Sepsis, characterized by generalized inflammation in response to infection, is estimated to affect greater than 650,000 patients in the United States per year and is responsible for over 120,000 in-hospital deaths [1]. Sepsis-related mortality has historically been attributed to an overwhelming *pro*-inflammatory response to infection that leads to multi-organ failure. However, it is now thought that a prolonged *anti*-inflammatory resolution phase is responsible for the majority of sepsis-related deaths [2], [3]. Some survivors of sepsis may continue to be more vulnerable to secondary insults for months after the initial infection, which may be the reason that these patients have greater than expected long-term mortality [4]. However, it is unclear which sepsis survivors are at greatest risk of subsequent infections and death.

Animal models of sepsis demonstrate that hosts with immune dysfunction exhibit altered inflammatory responses to infection [5]–[7]. Thus, we hypothesized that patients with immune dysfunction would be at high risk for death from secondary insults. Patients receiving myelosuppressive chemotherapies, immune-modulating agents for chronic inflammatory conditions or after organ transplantation, or people with immune disorders such as HIV infection are commonly thought of as being immunosuppressed [8]–[14]. These groups represent greater than one-third patients with sepsis in some reports [10], [12]. Because mortality from sepsis may be related to organism and source, we chose to focus on a single type of infection, *Staphylococcus aureus* bacteremia, which is common, typically causes a systemic inflammatory response, and affects people with and without prior immune dysfunction [15]. In addition, risk factors for mortality have been studied previously and hence can be controlled in a multivariable model [16].

## Materials and Methods

This study was conducted at the University of Chicago Medical Center (UCMC) in Chicago Illinois, a 547-bed, university-affiliated, urban teaching hospital. A microbiology database of all positive blood cultures from April 1, 2009 to April 1, 2012 was reviewed. Patients were included if there was at least one positive blood culture for *Staphylococcus aureus*, were ≥18 years of age, and required inpatient admission. Only the first episode of *S. aureus* bacteremia for a given patient was included in the analysis. The University of Chicago Institutional Review Board approved this study and waived the need to obtain informed consent. Patient information was anonymized and de-identified prior to analysis.

This study used a retrospective cohort design. All data were abstracted from patients' electronic medical records, which included nursing documentation, laboratory values, microbiology data, physician notes, radiology reports, and hospital discharge summaries. The reference data point for this study was the first day of positive blood cultures for *S. aureus*. The primary outcome variables were 30-day mortality and 31 to 90-day mortality. When these outcomes could not be determined from the medical records, the Social Security Death Index was reviewed.

The primary predictor variable of interest was prior immune dysfunction defined *a priori* as at least one of the following: an active hematological malignancy, an active solid malignancy requiring chemotherapy within the previous 60 days, a hematological or solid organ transplantation requiring immune-modulating medications, HIV infection, or chronic inflammatory condition and receiving immune-modulating medications within the previous 60 days. The minimal dose of corticosteroids considered to be immune modulating was 20 mg of prednisone daily or equivalent for one month. Other recorded baseline characteristics were age, gender, race, and comorbid conditions.

The index *Staphylococcus aureus* infection was categorized according to methicillin resistance of the *S*. *aureus* isolate and acquisition of the infection in a healthcare vs. community setting [17]. The initial antibiotic choice was deemed appropriate if the organism was susceptible to the antibiotic under in vitro testing and if the antibiotic was administered within 24 hours of the first positive blood culture. It was recorded whether an infectious disease team was consulted. For patients who survived longer than 14 days, antibiotic duration was categorized as either two weeks or greater than two weeks. Culture-free days were defined as the number of days the patient survived in the first 30 with negative cultures. A removable source was defined by a positive quantitative culture of a device for *S. aureus* after its removal. Endocarditis was defined by the modified Duke's criteria [18]. Severity of illness was determined by the Sequential Organ Failure Assessment (SOFA) score, which was calculated on the first day of positive blood cultures [19]. For patients who survived 30 days, inpatient infections in the next 60 days that occurred at the UCMC were recorded. Infections were categorized as bacterial based on the United States Center for Disease Control (CDC) definition [20]. Infections were categorized as fungal if they met “definite” or “probable” criteria based on an International Consensus Definition [21].

Continuous variables were reported as medians with 25th and 75th percentiles. The Student's t-test, the Mann–Whitney U test, or the Chi-square tests were used in bivariate testing, as appropriate, to determine if there were significant differences between groups. Logistic regression analysis was used to identify risk factors that were independently associated with 30-day mortality. Prior immune dysfunction was the primary variable of interest and was kept in the model. All other variables related to the infection and/or were plausibly linked to the outcome were initially placed in the model. A backward selection procedure was then employed to determine a final model. A Kaplan-Meier survival function and Cox proportional hazard model were used to examine the influence of prior immune dysfunction on 31 to 90-day mortality. All tests were two-sided and a p-value ≤0.05 was considered to indicate statistical significance. All analyses were performed with STATA 12.1 (StataCorp, College Station, TX).

## Results

There were 237 adult inpatients with *S*. *aureus* bacteremia at UCMC between April 1, 2009 and April 1, 2012, of whom 71 (30%) had prior immune dysfunction. The 30-day mortality was 23% and 31 to 90-day mortality was 12%. There were eight patients censored by day 30 and 13 patients censored by day 90. The most common sources were bacteremia without focus (44%), indwelling device (21%), and skin or soft tissue (13%).

Under bivariate analysis, increased age and SOFA score were associated with greater mortality at 30 days; culture-free days and infectious disease consultation were associated with *improved* mortality at 30 days (Table 1). These four factors remained statistically significant under multivariable analysis (Table 2). Prior immune dysfunction was not significantly associated with 30-day mortality (OR 1.54, 95% CI 0.57–4.16, p = 0.39).

**Table 1:** Characteristics of 229 patients with *S. aureus* bacteremia, comparing in bivariate analysis those who were alive and deceased 30 days after blood-stream infection.

	Alive at 30 days	Died at 30 days	p value
	(N = 177)	(N = 52)	
Age (median [25–75 percentile])	55 [41, 66]	62 [53, 76]	p<0.001
Male N (%)	97 (55)	27 (52)	p = 0.71
African-American N (%)	120 (68)	35 (67)	p = 0.95
Prior Immune Dysfunction N (%)	57 (32)	13 (25)	p = 0.32
Diabetes N (%)	56 (32)	13 (25)	p = 0.36
End-Stage Renal Disease N (%)	54 (31)	13 (25)	p = 0.44
Congestive Heart Failure N (%)	32 (18)	9 (17)	p = 0.90
Solid Malignancy without Chemotherapy N (%)	12 (6.8)	5 (9.6)	p = 0.49
Methicillin Resistant *S*. *aureus* N (%)	88 (50)	29 (56)	p = 0.44
Healthcare-associated or Hospital Acquired Infection N (%)	142 (80)	44 (85)	p = 0.48
Appropriate Initial Antibiotics N (%)	157 (89)	44 (85)	p = 0.43
Infectious Disease Consultation N (%)	131 (74)	22 (42)	p<0.01
At least two weeks of Intravenous antibiotics N (%)	104 (59)	9 (69)*	p = 0.46
SOFA Score (median [25–75 percentile])	4 [2], [6]	8 [6], [13]	p<0.0001
Culture-Free Days (median [25–75 percentile])	28 [26], [29]	23 [0, 29]	p<0.001
Removable Source N (%)	45 (25)	7 (13)	p = 0.07
Endocarditis N (%)	13 (7.3)	8 (15)	p = 0.08

* Denominator is number of patients who died between days 14–30.

**Table 2:** Multivariable model of factors associated with 30-day mortality among 229 patients with *S*. *aureus* bacteremia.

	OR	95% CI	p value
Age	1.05	1.02–1.08	p<0.01
Culture-Free Days	0.91	0.86–0.96	p<0.01
SOFA Score	1.37	1.20–1.56	p<0.001
ID Consultation	0.30	0.12–0.75	p = 0.01
Prior Immune Dysfunction	1.54	0.57–4.16	p = 0.39

During days 31 to 90, 11 patients (20%) with prior immune dysfunction and only 10 patients (8.6%) without prior immune dysfunction died (OR 2.59, 95% CI 1.03–6.53, p = 0.04). Table 3 shows the timing and frequency of death by type of prior immune dysfunction. Patients with prior immune dysfunction had similar risks of death during days 31 to 90 compared to days 0 to 30 (20% vs. 19%, p = 0.87), whereas patients without prior immune dysfunction had a lower risk of death in this later time period (8.6% vs. 25%, p<0.01). Figure one displays day 31 to 90 Kaplan-Meier survival functions categorized by presence or absence of prior immune dysfunction. For the 11 patients with prior immune dysfunction who died during days 31–90, one died from progressive chronic disease, three died from sepsis, and the cause was unknown for seven patients. For the 10 patients without prior immune dysfunction who died during this time period, one died from progressive chronic disease, five died from sepsis, and the cause was unknown for four patients.

**Figure 1:**
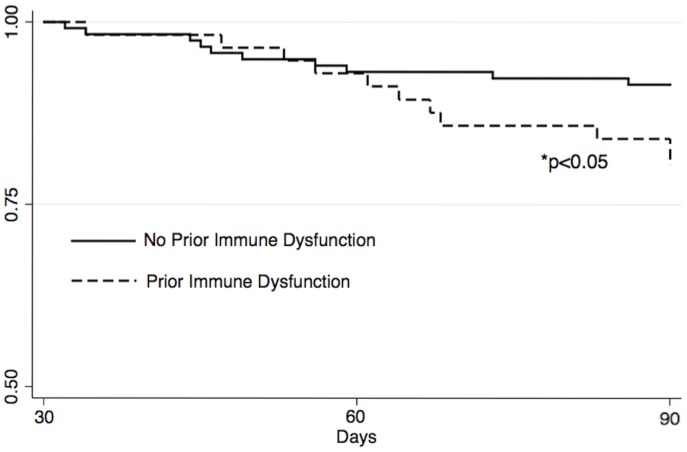
Day 31 to 90 survival functions by presence or absence of prior immune dysfunction.

**Table 3:** Timing and frequency of death by type of prior immune dysfunction.

		Died < 30 days	Died 31–90 days
		[N (%)]	[N (%)]
No Prior Immune Dysfunction [N = 159]*		39 (25)	10 (8.6)
Prior Immune Dysfunction [N = 70]		13 (19)	11 (20)
	Solid Malignancy on Chemo [N = 21]	4 (19)	4 (24)
	Hematological Malignancy [N = 17]	4 (24)	3 (25)
	Solid Organ Transplant [N = 8]	0 (0)	2 (25)
	Inflammatory Condition [N = 12]	2 (16)	2 (20)
	HIV [N = 12]	3 (25)	0 (0)

* [N] reflects number of patients at risk with each group at 30 days. There were 5 patients who were censored between 31–90 days, one of whom had a prior immune dysfunction (hematological malignancy)

There were 25 patients (43%) with prior immune dysfunction compared to 33 patients (26%) without prior immune dysfunction who had subsequent infections noted in the medical record in days 31 to 90. The infectious pathogens are listed in Table 4. Of the 11 patients who had subsequent infections and died between 31–90 days, sepsis was the cause of death in 8 (73%). In a Cox-proportional hazard model controlling for age, there was a significant association between prior immune dysfunction and increased 31 to 90 day mortality (HR 2.44, 95% CI 1.01–5.90, p = 0.05) and a non-significant trend towards subsequent infections and increased 31 to 90 day mortality (2.12, 95% CI 0.89–5.07, p = 0.09) (Table 5). Figure two displays day 31 to 90 Kaplan-Meier survival functions categorized by presence of prior immune dysfunction and subsequent infections.

**Figure 2:**
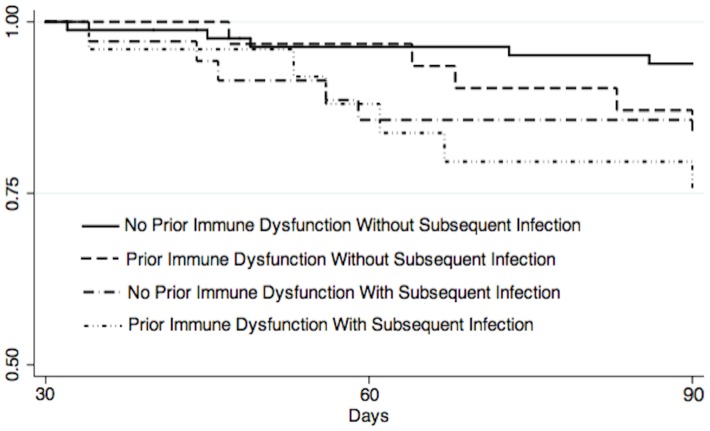
Day 31 to 90 survival functions by presence or absence of prior immune dysfunction and subsequent infections.

**Table 4:** Subsequent infectious pathogens among 30-day survivors of *S. aureus* bacteremia.

	Prior Immune Dysfunction	No Prior Immune Dysfunction
	(N = 25)	(N = 33)
*Staphylococcus* sp N (%)	5 (20)	9 (27)
*Enterococcus* sp N (%)	1 (4.0)	3 (9.1)
Gram Neg. Rod N (%)	4 (16)	7 (21)
*C.* Difficile N (%)	4 (16)	2 (6.1)
Fungal N (%)	1 (4.0)	3 (9.1)
Unknown pathogen N (%)	10 (40)	9 (27)

**Table 5:** Cox proportional hazard model investigating factors associated with mortality days 31–90 after *S*. *aureus* bacteremia, controlling for effects of age.

	HR	95% CI	P value
Age	1.04	1.01–1.07	p = 0.01
Prior Immune Dysfunction	2.44	1.01–5.90	p = 0.05
Subsequent Infection	2.12	0.89–5.07	p = 0.09

## Discussion

In this study, we report that among patients with *S*. *aureus* bacteremia, clinical immune dysfunction is associated with 31 to 90-day mortality, but not 30-day mortality. The fact that prior immune dysfunction does not increase the risk of short-term mortality from a serious bacterial infection likely runs contrary to the intuition of many clinicians. To our knowledge, only one study has used a similar definition of immunosuppression to ours and found an association with 30-day mortality after *S*. *aureus* bacteremia [15].

We report for the first time that the risk of death for patients with prior immune dysfunction does not decrease over days 31 to 90 as it does for patients without prior immune dysfunction. This interesting finding provides insight into the types of patients who are highest risk for death in the months after sepsis. The outcome variable of 31 to 90 day mortality is not commonly reported in sepsis studies, but is likely an important one given the current paradigm of sepsis pathophysiology. Heightened awareness for signs of clinical decompensation among survivors of sepsis with prior immune dysfunction may improve outcomes.

Previous studies on *S*. *aureus* bacteremia have reported short-term mortality rates of 20% [15], [22] and a 31 to 90 day mortality of approximately 10% [23], which are similar to our findings of 23% and 12% respectively. Immunosuppressed patients accounted for 20–25% of the population with S. *aureus* bacteremia in prior reports [15], [22]. Thirty percent of our cohort met our definition of prior immune dysfunction. The factors associated with greater 30-day mortality in our cohort: age [22], [24], [25], persistent bacteremia [26], [27] and severity of illness [28], [29] have been identified by other investigators as predictors of outcome. Also similar to previous investigators, we found infectious disease consultation to be associated with lower mortality [15], [30], [31]. We hypothesize that patients in our cohort who received infectious disease consultations were managed more in accordance with evidence-based guidelines. Other factors such as methicillin resistance and other co-morbid conditions have not been as consistently associated with survival [16]. Investigators have reported that higher comorbidity index scores are associated with mortality from *S*. *aureus* bacteremia [32]. Our results suggest that conditions that result in immune dysfunction are the primary comorbidities driving this association.

Although patients with prior immune dysfunction represent a large subpopulation with sepsis, there have been relatively few studies focusing on this as a prognostic variable. In a study of over 1000 patients with sepsis, not limited to *S. aureus* bacteremia, immunosuppressed patients had greater mortality at 28 days (adjusted RR 1.62, 95% CI 1.38–1.91) [12]. In an international, observational study of ICU patients with infections, immunosuppression was one of the factors associated with mortality after discharge from the ICU [8]. We build on the results of this study by demonstrating a similar finding for patients with a single, common infection type and by examining subsequent infections as risk factors for long-term mortality.

While poor prognosis of chronic disease likely plays a role in the increased long-term mortality for patients with prior immune dysfunction and *S. aureus* bacteremia, we hypothesize that a unique immunological response to blood-stream infection also contributes. Animal models of sepsis demonstrate that hosts with prior immune dysfunction exhibit altered inflammatory responses to sepsis [5], [7]. In a recent study, a group of pigs were iatrogenically immunosuppressed with prednisone, cyclosporine, and mycophenolate and then underwent cecal ligation and puncture. This group exhibited a blunted pro-inflammatory IL-6 response and significantly increased anti-inflammatory IL-10 response compared to controls [6]. Elevated IL-10 levels have been associated with increased mortality after *S. aureus* bacteremia [33] and among general populations with sepsis [34]. Further exploration of the immune response to *S. aureus* bacteremia in patients with and without prior immune dysfunction is clearly warranted.

Our study has the following limitations: because only 21 patients died between 31 to 90 days, we were limited in the number of covariates we could include in a Cox proportional hazard model. However, there are no specific variables that have definitively been shown to be associated with death in the months after severe infection. Second, we were only able to capture subsequent infections that occurred at our institution. However, it seems likely that most patients would return to the same institution for care within 90 days of a severe infection. Third, there is no consensus definition of “prior immune dysfunction;” we used a definition that is clinically relevant and is similar to one used by other investigators [8]–[14]. Finally, we could not consistently determine cause of death. It is possible that chronically ill patients are more likely to die *with* infections, not *because* of infections. However, of the 11 patients with subsequent infections who died during days 31–90, 74% died from sepsis, which suggests that most of these infections are clinically important. While we did not examine survival of patients with prior immune dysfunction without infections, bacteremia has previously been reported to be a poor prognostic factor in immunosuppressed populations [35], [36]. Still, our conclusions might be stronger if we knew whether deaths by day 90 were directly due to the development of new infections or progression of chronic diseases for all patients. In total, these findings and limitations support the need for a prospective study.

## Conclusions

Guidelines for the management of sepsis emphasize early interventions, and thus have had the greatest impact on early mortality. There are no interventions to improve long-term outcomes from sepsis, likely because it is unclear who is highest risk for death in the months after sepsis. In this study, we provide evidence that patients with prior immune dysfunction are at higher risk of death than are other patients during days 31 to 90 after *S*. *aureus* bacteremia, perhaps because of increased risk of new infections. We suggest future research endeavors focusing on the immune response to sepsis specifically in this population.
